# Mechanoresponsive Self‐Assembled Perylene Bisimide Films

**DOI:** 10.1002/chem.202001805

**Published:** 2020-07-08

**Authors:** Victoria Adams, Joseph Cameron, Matthew Wallace, Emily R. Draper

**Affiliations:** ^1^ School of Chemistry University of Glasgow Glasgow G12 8QQ UK; ^2^ School of Pharmacy University of East Anglia Norwich NR4 7TJ UK

**Keywords:** flexible, organic electronics, perylene, self-assembly, smart materials

## Abstract

In this work, self‐assembled amino‐acid appended perylene bisimides (PBIs) have been studied that when processed into thin films change their resistivity in response to being bent. The PBIs assemble into structures in water and form thin films upon drying. These normally delicate thin films can be tolerant to bending, depending on the aggregates they form. Furthermore, the films then reversibly change their resistivity in response to this mechanical stimulus. This change is proportional to the degree of bending of the film giving them the potential to be used quantitatively to measure mechanical movement, such as in wearable devices.

## Graphical abstract

**Exploring how** the self‐assembly of three different perylene bisimide structures in solution has an effect on the final properties of a dried thin film. These structures were characterized by aligning under shear and under a magnetic field. It was found that how they dry into a film has a major effect on their use in flexible conductive materials and it was demonstrated how this resistivity is proportional to the angle they are being bent at.

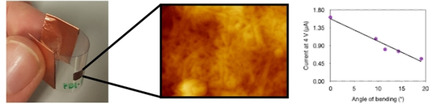

There is significant attraction towards using flexible conductive materials when designing “smart materials”, this is due to the potential of them being used for wearable electronics.^1^ Flexibility can be used to describe a variety of properties, such as, being bendable, stretchable, resilient or lightweight enough to facilitate movement.1b When referring to smart textiles these are; passive smart, which only sense the environment around them; active smart, have the ability to react to what they have detected; and very smart, can adapt and react based on the information they have collected. Such materials can be achieved by weaving conductive fibres,^2^ or using conductive ink on the desired substrate.^3^ Regardless of application and substrate these materials need to be processed in order to ensure flexibility, robustness and durability.^4^ This is an area where metal‐based materials have triumphed, as metals are highly conductive, durable and can be processed into fibres or nanoparticles for example.^5^ Development of strain responsive systems are core to such concepts as robotic skin, which require thousands of separate sensors packed onto a flexible substrate.^6^ They not only need to sense strain, but also pH, moisture and temperature, like real skin. This is not currently possible with metal based or Si‐based electronics. Organic field effect transistors have been suggested as a possible solution.^7^ Organics have the advantage of being able to be simply processed, such as by screen printing, and so can cover large areas with thousands of separate sensors easily.^3^, ^8^ There are however problems associated with organics, like, durability, longevity, and degradation. More recently there are now examples of organics matching and now surpassing the outputs of their metal alternatives such as in OLEDs,^9^ H_2_ evolution,^10^ and solar cells.^11^ A group of materials we have significantly investigated the chromic, and semi‐conductor behaviour of are amino acid appended perylene bisimides (PBIs). We have found them to be thermally robust, air stable, moisture insensitive, processable with a remarkably long‐lived radical species.^12^ Other groups have used PBIs for a variety of sensing, (blood oxygen,^13^ amine,^14^ temperature^15^ and pH sensors^16^) showing they could be the ideal candidates for robotic skin.^17^ One drawback of these materials is inflexibility. When PBI based materials are used in flexible films, they are often combined with polymers, polymerised themselves or appended to a polymer in order to achieve flexibility.^18^ When conductivity was measured in these systems the effect of strain or stress was not carried out. In an example where the resistance was measured, the PBIs were assembled with graphene and used a photo sensor. These materials displayed an increase in resistivity as the material was bent to 5 mm.^19^ It is however difficult to compare between different materials due to the differences in sample preparations and experimental set up.

Here, we show how the structure of simply functionalised PBIs in water affects the flexibility of the thin films formed on a flexible substrate, and how bending of the film affects the resistivity of material. Initially three different amino acid functionalised PBIs were investigated, l‐histidine (**PBI‐H**), l‐alanine (**PBI‐A**) and l‐phenylalanine (**PBI‐F**) (Figure 1).

**Figure 1:**
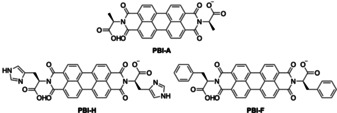
Chemical structures of the PBIs.

From previous work, we saw that **PBI‐A** and **PBI‐H** gave the largest photo‐response, (12 % and 8 % radical, respectively) and so were suitable to test. Whereas **PBI‐F** showed little radical anion (less than 1 %), so would be an interesting comparison.12a, 12b At 0.5 wt % in water these PBIs assemble, as characterised by small angle neutron scattering and rheology.12b **PBI‐A** fit to a flexible cylinder model, with radii of 51 Å, whereas **PBI‐F** and **PBI‐H** fit to an elliptical cylinder with radii of 11 Å and 72 Å respectively. These structures persist upon drying into to a thin film which show photo‐responsive behaviour to UV light12a, 12b allowing us to investigate whether the structure effects the flexibility. Before analyzing the response of the thin films, the structures in solution were examined. We used shear induced alignment, to image the samples under cross polarised light whilst a shear force is applied (Figure 2).^20^ Alignment of the structures result in a Maltese cross, with more anisotropic materials appearing brighter in the images. Using a shear rate of 1000 s^−1^, **PBI‐A** showed the most alignment, **PBI‐H** showed some alignment and **PBI‐F** showed no alignment. This difference in alignment on this length scale shows that the aggregates formed from each of the perylenes are different. Structures formed from **PBI‐A** must be sufficiently long, persistent or numerous enough to align under shear, as are structures from **PBI‐H**. The intensity of alignment may suggest that the lengths of aggregates are longer in **PBI‐A** than **PBI‐H**. However, **PBI‐F**’s lack of alignment indicates smaller or few structures in solution. These agree with the previous SANS data collected.

**Figure 2:**
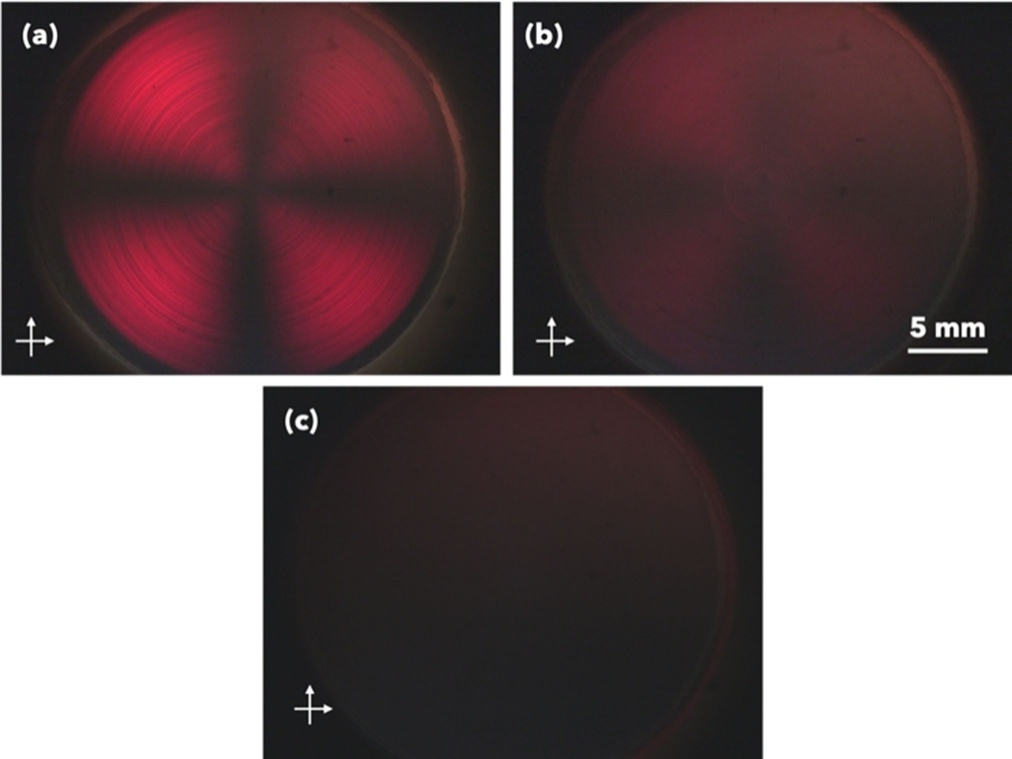
Shear induced alignment imaged under cross polarised light at 1000 s^−1^ (a) **PBI‐A**, (b) **PBI‐H** and (c) **PBI‐F**.

Next, we used ^23^Na NMR spectroscopy to probe the size and charge of the structures and whether the structures can align under the magnetic field of the spectrometer. Following previous work, we again observe the alignment of **PBI‐A** structures in the magnetic field which causes a residual quadrupolar coupling of the ^23^Na resonance to be observed (Figure 3).^21^ The *T*_1_ and *T*_2_ relaxation times of ^23^Na are also reduced relative to a solution in the absence of PBI due to the interaction of ^23^Na^+^ with the structures present.^22^ Lorentzian deconvolution of the spectra yields a T_2_ of 38 ms for the central peak and 3 ms for the quadrupolar satellites. The separation between the quadrupolar satellites is 211 Hz. For comparison, the *T*_2_ of ^23^Na^+^ in small molecule solution is singular at ca. 55 ms.^21^ In contrast to **PBI‐A**, for **PBI‐F** the ^23^Na resonance is a single Lorentzian peak with a fitted T_2_ relaxation time of 23 ms. The ^1^H resonances of **PBI‐F** are also broad suggesting a degree of aggregation (Figure S4). However, the ^2^H resonance of D_2_O does not exhibit quadrupolar splitting, again suggesting the absence of any alignment in the magnetic field. We conclude that **PBI‐F** is aggregated to some extent, but the structures are too small to exhibit either magnetic or shear alignment. **PBI‐H** exhibits a splitting of the D_2_O resonance indicating alignment of the structures with the spectrometer field. The quadrupolar satellite peaks of ^23^Na^+^ are broadened beyond detection while the central peak yields a fitted *T*_2_ value of 6 ms.

**Figure 3:**
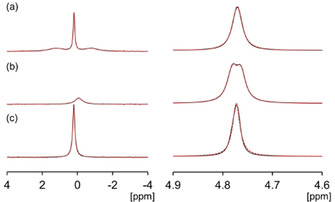
^23^Na (left) and ^2^H (right) NMR spectra (D_2_O) of (a) **PBI‐A**, (b) **PBI‐H** and (c) **PBI‐F**. Lorentzian fits to spectra are shown as red dashed lines.

Atomic force microscopy (AFM) was used to probe the structures that may persist or form upon drying. The full experimental details for AFM analyses are presented in the Supporting Information. The films were studied on both glass and treated plastic and the general topographies were consistent between the two substrates (Figure S5). **PBI‐A** and **PBI‐H** both showed bunched, thin fibrous structures upon drying, whereas **PBI‐F** showed a rough surface with no defined structures, agreeing with the NMR and shear alignment data. **PBI‐A** showed a generally more uniform surface compared to **PBI‐H**, with a larger number of gaps appearing between the fibres in the **PBI‐H** films. This can be shown by calculating the number of holes and area of the films. For example, in the plastic substrates, PBI‐A showed 27 holes with a total area of 204.9 μm^2^ whilst PBI‐H had 35 holes with a total area of 211.2 μm^2^. It would be expected that the more uniform **PBI‐A** surface will contribute to improved photoconductivity. Similarly, the more complete coverage of the **PBI‐A** film would likely be less prone to cracking upon bending.

To test the flexibility of the PBI films, the PBIs were drop cast onto PVC plastic to allow for bending of the substrate. The plastic was plasma treated to give better wettability (Figures S6 and S7) and to improve the contact of the material and the substrate. The better contact angle allows for a more uniform distribution of the material and better‐quality films. The UV/Vis absorption spectra for samples cast on glass compared to plasma treated plastic showed no difference in the absorption of the material (Figure S8) making our data comparable to that previously collected.

Current–voltage (IV) plots were collected for the films on plastic, in the dark and after irradiation with 365 nm light for 5 minutes. From previous work we know that these PBIs only respond to light <400 nm. All films showed ohmic contact with currents of less than 2 μA at 4 V. As seen with previous trends on glass **PBI‐A** (μA range) has the greatest response and **PBI‐F** the smallest (nA range), with **PBI‐H** being in between (Figure S9).

Upon inspection of the films under a microscope, **PBI‐F** appeared cracked, the reason for decreased current upon multiple measurements (Figure S10). **PBI‐H** and **PBI‐A** showed a continuous film (Figures S11 and S12). **PBI‐F** films were not suitable to be used for the rest of the study due to the degradation in the material. This could be due to **PBI‐F** being more hydrophobic than the other molecules, so even on the treated plastic they did not form a robust film, or the structures formed are less likely to form a uniform film.

Bending of the films was tested using a series of 3D‐printed holders to ensure that each of the films were bent in the same way and controllably (Figure 4 a and Figures S1–S3). Angles of 0.0°, 9.5°, 11.5°, 14.3° and 19.1° were tested, with 0.0° being the least bent and 19.1° the most. The effect of the degree of bending on the resistance of the films was carried out under constant irradiation with 365 nm. From chronoamperometry, the current stabilized after 5 minutes. From previous EPR and UV/Vis absorption measurements, it could be assumed the sample was saturated with radical anion so change in the current is a result of bending. A blank set up was tested to ensure that results were from the films not the substrate itself (Figure S13). **PBI‐H** films showed no ohmic contact for all angles greater than 0° (Figure S14). After the measurement the microscope showed the films were covered in small cracks (Figure S15). The **PBI‐H** films were not flexible, and these cracks disrupted the contiguous pathway stopping the conductivity. **PBI‐A** films did not show cracking under the microscope and kept ohmic contact during these measurements (Figure S16). Making them suitable for testing. Bending the films decreased the current, with the largest angle having the biggest effect (Figure 4 b, Figures S17 and S18 a) returning to around the original value upon being straightened (Figure S18 b). The films could be bent again to show a similar value to that of the first time it was bent (Figure 4 c and Figure S19).

**Figure 4:**
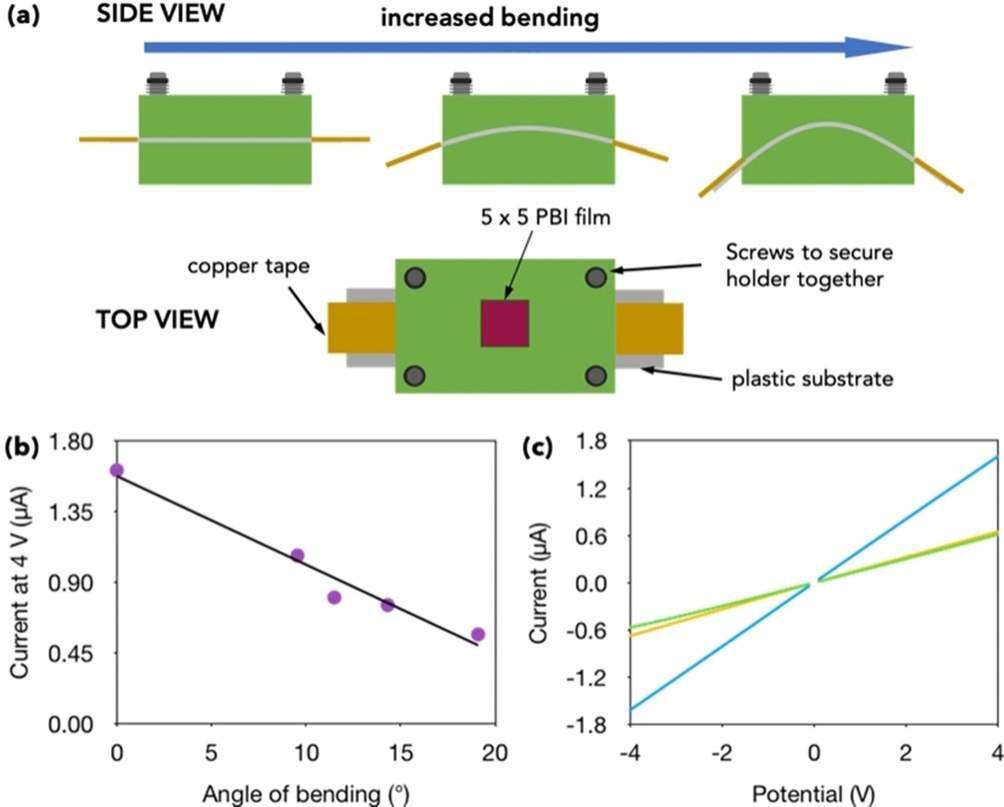
(a) Cartoon showing the film holders used for bending. (b) Current at 4 V whilst bending **PBI‐A film**. The solid line=line of best fit with R^2^=−0.988. (c) IV data showing the recoverability of the **PBI‐A** film. Blue data=0°, green data=19.1°, yellow data after the sample was straightened, then remeasured at 19.1°.

Encouragingly all the **PBI‐A** films showed a proportional decrease in current to the degree of bending, with a linear relationship, R^2^, between −0.98 and −0.95 (Figure S17). The **PBI‐A** films were then subjected to more vigorous bending at the largest angle. After 20 bending cycles fine cracks on the surface of the films started to appear but had no impact on the resistance of the films (Figure S20).

The differences between **PBI‐H** and **PBI‐A** upon bending could be due to the length of the fibres. From the SANS, NMR and alignment data we can assume that **PBI‐A** has longer structures than **PBI‐H**. It is therefore our hypothesis that with longer fibres, upon bending the **PBI‐A** fibres act like layers that can slide over each other, increasing the length of the film and therefore creating longer pathways for the current and increasing the resistivity.^23^ **PBI‐H** has shorter structures and bending creates gaps in the film. Moreover, AFM and microscopy images show that **PBI‐A** surface is generally more uniform than the surface of **PBI‐H**, so will be less prone to cracks forming as a result of bending.^24^ The residual stress from the substrate may be too much for the **PBI‐H** films and results in detrimental cracks.^24^ The lack of structures with **PBI‐F** results in a material without a contiguous film. Im et al. saw similar observations when working with macroscopically aligned fibrous PBIs, noting they had better mechanical properties and flexibility than isotropic, or non‐fibrous films.18b

In conclusion, we demonstrate a reversibly mechanoresponsive material from **PBI‐A** with a response proportional to the degree of bending, which would be suitable for a movement sensor. Unlike other PBI based examples, the PBI does not need to be assembled with polymers or graphene to be flexible. Furthermore, we have shown the ability to form these flexible films is determined by the structures present in solution. We believe the fibrous structures produce conductive films with the longer fibres being responsible for the flexible responsive behaviour.

## Conflict of interest

The authors declare no conflict of interest.

## Biographical Information

*Emily Draper is currently a Lecturer at the University of Glasgow, UK. She holds a Leverhulme Trust Early Career Fellowship and a Lord Kelvin Adam Smith Leadership Fellowship. She received her PhD from the University of Liverpool in 2015 working on gels, then continued on as a PDRA. She moved to Glasgow in 2016 as a PDRA before starting her Fellowships in 2017. Emily then started a Lectureship at Glasgow in 2018 before having her first child in 2019. Her research interests include organic electronics, chromic materials, self‐assembly, and combining computational and experimental based approaches for materials design. In her spare time, she likes visiting castles and bagging Munros*.

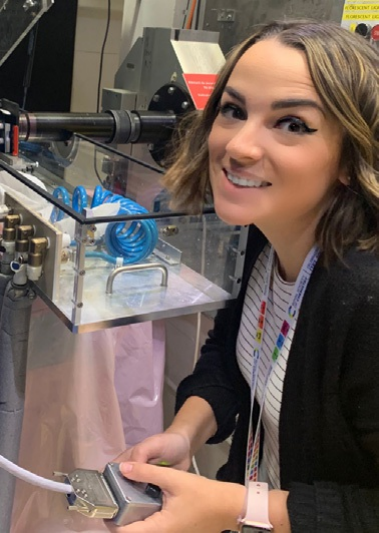

## Supporting information

As a service to our authors and readers, this journal provides supporting information supplied by the authors. Such materials are peer reviewed and may be re‐organized for online delivery, but are not copy‐edited or typeset. Technical support issues arising from supporting information (other than missing files) should be addressed to the authors.

Supplementary
