# Supplementary material for: Mechanoresponsive Self‐Assembled Perylene Bisimide Films
Source: Chemistry. 2020 Jul 8;26(44):9879–82. doi: 10.1002/chem.202001805 (PMC7522684; doi:10.1002/chem.202001805)
Supplement: Supplementary file 1 — Supplementary [file CHEM-26-9879-s001.pdf]

Chemistry–A European Journal

Supporting Information

Mechanoresponsive Self-Assembled Perylene Bisimide Films

Victoria Adams,[a] Joseph Cameron,[a] Matthew Wallace,[b] and Emily R. Draper*[a]

Mechanoresponsive Self-Assembled Perylene Bisimide Films

Supporting Information

Victoria Adams,[a] Joseph Cameron,[a] Matthew Wallace[b] and Emily R. Draper*[a]

[a]School of Chemistry
University of Glasgow
Joseph Black Building, Glasgow, UK, G12 8QQ

[b] School of Pharmacy
University of East Anglia
Norwich Research Park, Norwich, UK, NR4 7TJ

*E-mail: Emily.Draper@glasgow.ac.uk

Experimental Procedures S2
Supplementary Figures S6
References S15

Experimental Procedures

Stock Solution preparation. PBI-A, **PBI-H** and **PBI-F** were synthesised in house as previously reported.[1] All samples were prepared at a concentration of 5 mg/mL in water. 1 molar equivalent of 0.1M NaOH was used to disperse the PBIs into solution and made up to concentration with deionised water. These were allowed to stir with a magnetic stirrer until all the power was visibly dissolved, typically 16 hours.

Plasma Treatment. A Diener electronic femto plasma-surface-technology machine was used with an evacuated chamber before oxygen gas was added to create oxygen plasma. This set up was used to oxygenate the surface of plastic to make it more hydrophilic and allow for better adhesion of the PBI solution onto the surface. Plastic was placed on to the electrode in a single layer and the electrode was placed back into the machine, ensuring it was lying flat. Operation of the machine was maintained at full power, around 98%, and the oxygen plasma treated the sample for 4 minutes before the plastic was removed.

Film Preparation. Thin films were prepared on a Lyreco PVC clear covers that were cut to the required size. 20 µL of the required solution was dropped using a pipette inside a 5 x 5 mm sticky tape mask and leaving to air dry. Once dried, the mask was removed to produce a 5 x 5 mm square. Two silver electrodes were added to the edge of the sample using silver paste (Agar Scientific, Acheson Silver Electrodag 1415), ensuring that full contact was made to opposite sides of the sample square (Figure S1). Two pieces of copper tape (RS components) were then stuck to the silver electrodes so that they could be attached to the potentiostat.

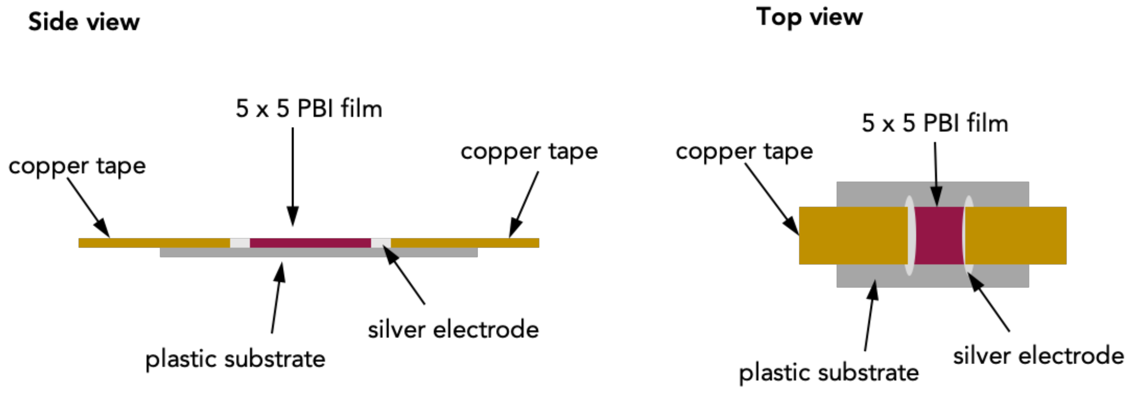

Figure S1. Cartoon showing how the films were set up for the conductivty measurements from the side (left) and from above (right).

pH Measurements. pH measurements were performed using a FC200 pH probe (HANNA Instruments) with a 6 mm x 10 mm conical tip. The accuracy of the pH measurements is quoted as ± 0.1.

^{1}H, ^{2}H and ^{23}Na NMR. The experiments are based on previous work, where we have shown that 2NapFF and **PBI-A** align in a magnetic field, which leads to the residual quadrupolar coupling effects seen here.[2,3] This alignment occurs in a 5 mm NMR tube once inserted into the spectrometer and is field dependent. NMR spectra were acquired using a Bruker 400 MHz (^{1}H) spectrometer equipped with a broad band probe operating at 105.84 MHz for ^{23}Na. ^{23}Na spectra were acquired in 256 scans using a 12 μs 90-degree pulse, a signal acquisition time of 0.3 s, a relaxation delay of 0.1 s and a sweep width of 120 ppm. ^{2}H spectra were recorded *via* the lock channel with 128 scans, a 200 μs 90-degree pulse, signal acquisition time of 2.0 s, a relaxation delay of 0.2 s and a sweep width of 10 ppm. ^{1}H spectra were acquired using the perfect echo WATERGATE sequence of Adams *et al.*[5] incorporating the double echo W5 sequence of Liu *et al.*[6] The acquisition time was set at 2.5 s. The delay between successive hard pulses of the selective pulse train was set at 333 μs corresponding to a 3000 Hz spacing between the null points. The relaxation delay was set at 1 s. 4 dummy scans and 16 scans were acquired. 1 mL of samples of each sample was used for these experiments and prepared as described above with 0.1 vol% D_2O added as a probe for ^{2}H NMR while 0.003 vol% THF was added to provide an internal reference signal. All spectra were calibrated to ^{1}H THF at 1.88 ppm.[7]

Spectra were processed in Bruker Topspin 3.6.2. ^{23}Na spectra were processed with an exponential line broadening factor (LB) of 3 Hz. The linewidths at half height of ^{23}Na ($\Delta\nu_{1/2}$) were obtained by Lorentzian deconvolution. T_2 relaxation times of ^{23}Na were obtained using Equation S1:[8]

$$T_2 = \frac{1}{\pi\left(\Delta\nu_{1/2} - \mathrm{LB}\right)} \qquad \text{(Eq.S1)}$$

The contribution to the linewidth from the magnetic field inhomogeneity can be neglected as analysis of the THF resonances confirmed it to be less than 2 Hz for ^{1}H (Figure S4b).

UV-vis Absorption Spectroscopy. Solid UV-Vis absorption data was obtained with an Agilent Cary 60 UV-vis spectrophotometer. Samples were cast onto the plastic used for the photoconductivity measurements. Data was collected between 350 – 800 nm at 200 nm/min.

Optical Microscopy. Optical microscope images were collected using a Nikon Eclipse LV100 microscope with a Nikon Plan ELWD 50x/0.60 or x5 lens attached to an Infinity2-1C camera.

Shear Induced Alignment. Shear alignment data was collected on an Anton Paar Physica 302 rheometer fitted with the RheoOptics accessory with cross polarisers. Camera Lumenera Lu165c: 12-bit, 1392 x 1040 pixels, 2/3" CCD-Sensor, USB 2.0

with an Edmund Optics Worldwide white Mi-LED fibre optic LED illuminator with iris light source.

Alignment was induced using a 25 mm parallel plate at 1000 s^{-1} at a gap of 0.1 mm. Samples were pipetted onto the plate (0.5 mL) and the plate lowered onto the solution. The sample was trimmed before the measurement was started and checked for any bubbles on the camera. If any bubbles were present the solution was replaced. The tests were only started when there were no bubbles present, the presence of bubbles interferes with the images. Samples were allowed to shear for 5 minutes, but alignment if it happened was almost instantaneous and so images were taken using the Luminera software after 1 minute. When the shear was stopped, any sample that showed alignment quickly de-aligned as we have previously reported.

Atomic Force Microscopy. Thin films were prepared were prepared as described above, either on plastic or glass for testing. The films were studied using a Bruker Innova atomic force microscope. Each image consists of 512 lines with 512 points per line. The scan rate was 1.0 Hz for the experiments and the scan area of the plots is 20 × 20 µm unless otherwise stated. The images were analysed using the NanoScope Analysis 1.5 program by Bruker. The default bow remove processing for the software was carried out to level the plots.[9] Hole analysis was carried out using WsXM 5.0 Develop 9.4 software with holes defined as having a minimum area of 0.0061 μm^2 at below 50% of the maximum height.

Photoconductivity Measurements. Photoconductivity measurements were performed using a Palmsens Potentiostat using a two-electrode measurement. Linear sweep measurements were recorded from -4 V to 4 V at a scan rate of 0.05 V/s. A 'dark' base line reading was collected before any irradiation happened. Samples were irradiated at 1 cm away from the sample 365 nm LED powered with an ISO-TECH IPS303DD DC power supply, which operated at a constant current of 0.7 A for 5 minutes, or until the amount of radical stopped increasing. All measurements and samples were collected at least in triplicate to ensure reproducibly of the results.

Bending experiments. OpenSCAD was used to design the film holders which consisted of two pieces of plastic that fit together on top of the films on plastics as described above. The top piece of the holder had a square cut out to allow the film to be irradiated. The curvature radius was changed to get holders (Figure S2 a and b) with a different curvature allowing the films to be bent to various degrees. The degree of bending was calculated using Equation S2. Five different angles were tested, 0.0°, 9.5°, 11.5°, 14.3° and 19.1°.

(Eq.S2)

$$\theta = \frac{arc\ length}{circumference} 360°$$

These were exported to .STL format in order to print using a 3D printer (Figure S2c).

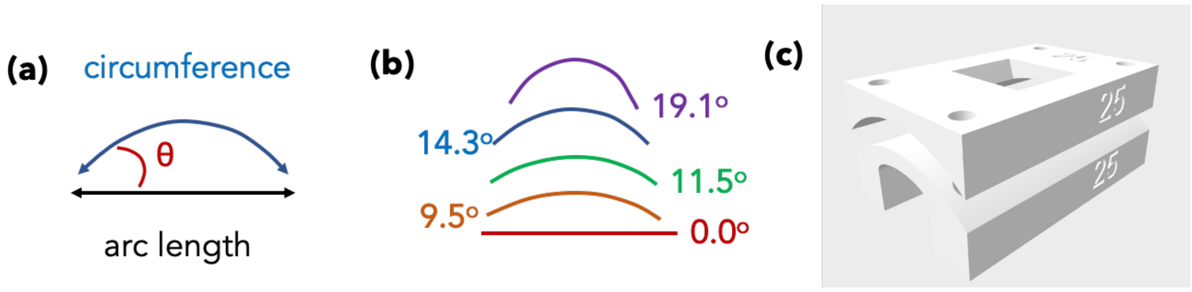

Figure S2. (a) Cartoon showing how the degree of bending were controlled and calculated. The shorter the arc length the more bent the film and the larger the angle (b) a cartoon showing what the bending angles of the film look like (c) SCAD drawing from the 9.5° film bender.

The holders were fixed together with screw to hold the film in place during the measurements and allowing the films to be removed without damage (Figure S3). At least 9 repeats were carried out for each of the materials tested, however for the ease of reading of the document we have included 3 repeats, as they all showed the same behaviour, but had slightly different resistivities to start with, as they were all different films and we have found that there is some variability in the film resistivity to start with. The samples were irradiated for at least 10 minutes before measurements were carried out and were kept under constant 365 nm light to ensure they stayed conductive. The films were bent in order of increasing curvature and then were left to completely recover in the dark then re-irradiated and the data repeated to check recoverability. Randomly selected angles were then checked to ensure reliability of the results.

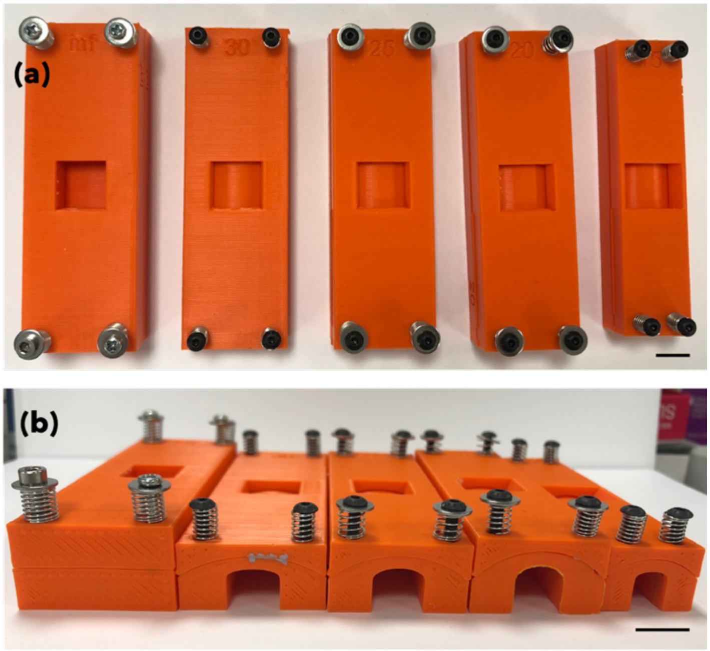

Figure S3. Photographs of the 3D printed film holders left to right, 0.0°, 9.5°, 11.5°, 14.3° and 19.1° viewed from (a) the top and (b) the side. Scale bar represents 1 cm.

Supplementary Figures

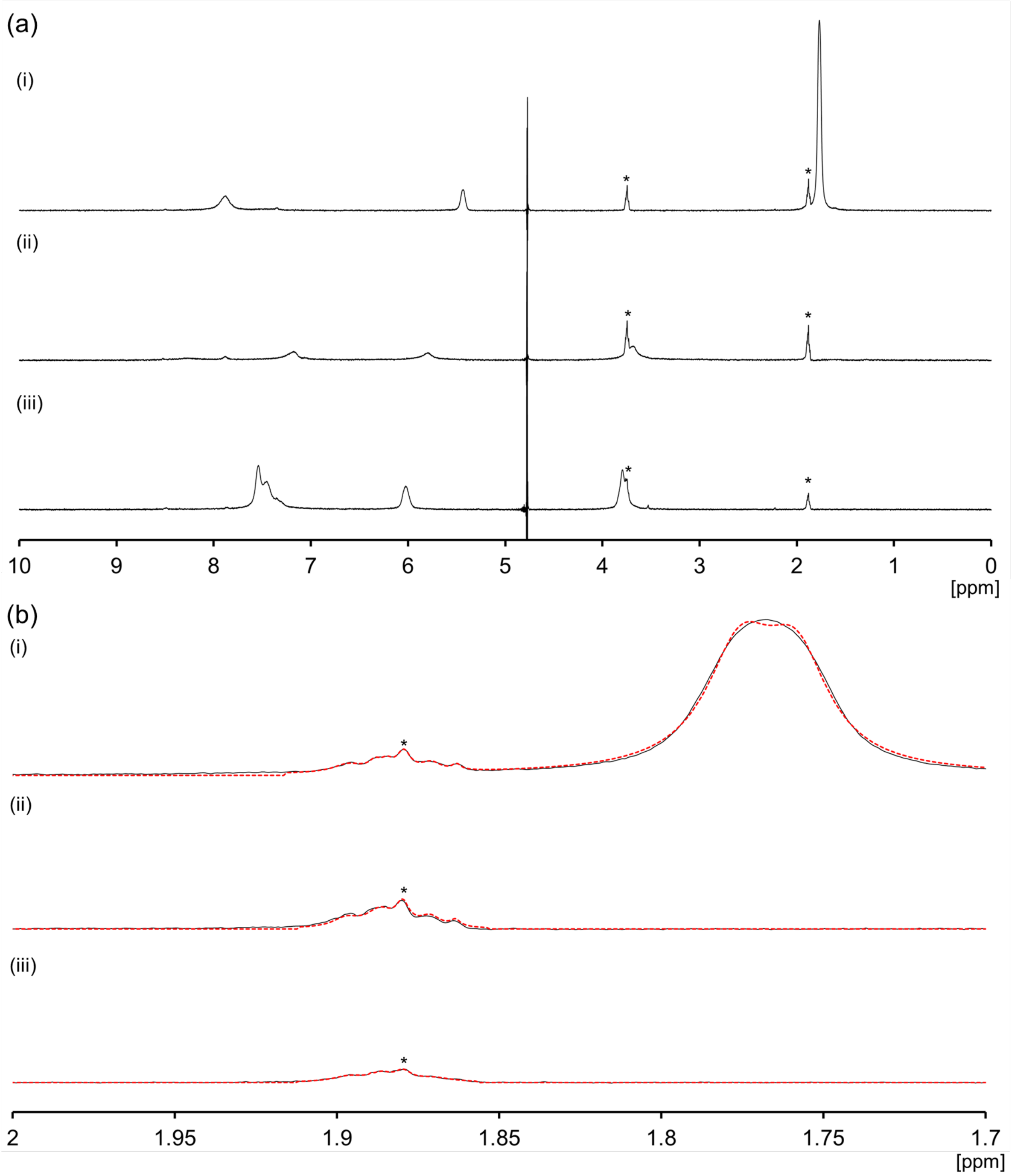

Figure S4. ^{1}H NMR spectra of (i) **PBI-A**, (ii) **PBI-H** and (iii) **PBI-F**. The sharp peaks marked * belong to 0.003 vol% THF. Other resonances belong to PBI. Spectra have been processed with an exponential line broadening factor of 0.3 Hz. Full spectra (a) and expansions to show upfield THF resonance (b). The dashed red lines on (b) are Lorentzian fits to the spectra. The peak marked * has a fitted linewidth of 2 Hz in all samples, confirming that the ^{23}Na linewidths will be unaffected (< 0.5 Hz) by the magnetic field inhomogeneity and that THF does not interact strongly with the PBI structures. The broadness of the PBI resonances relative to THF indicates that the PBI are aggregated.

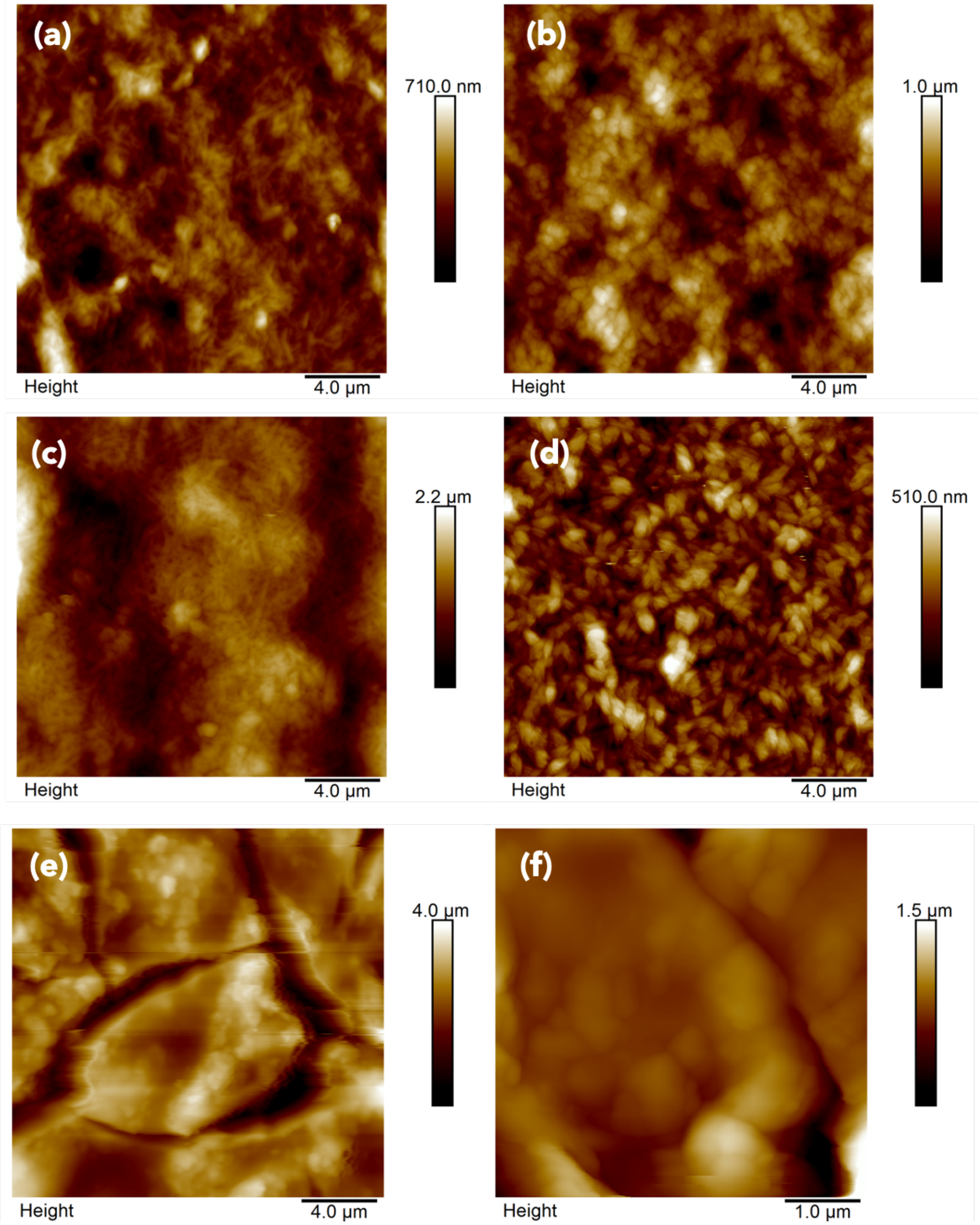

Figure S5. AFM images of films of **PBI-A** ((a) and (b)), **PBI-H** ((c) and (d)) and **PBI-F** ((e) and (f)). (a), (c) and (e) are on glass and (b), (d) and (f) are on treated plastic. All images are 20 × 20 µm scan size apart from (f) which is 5 × 5 µm. It was difficult to produce a clean AFM image of the PBI-F film on plastic substrate at a larger scan area due to the high roughness of the film.

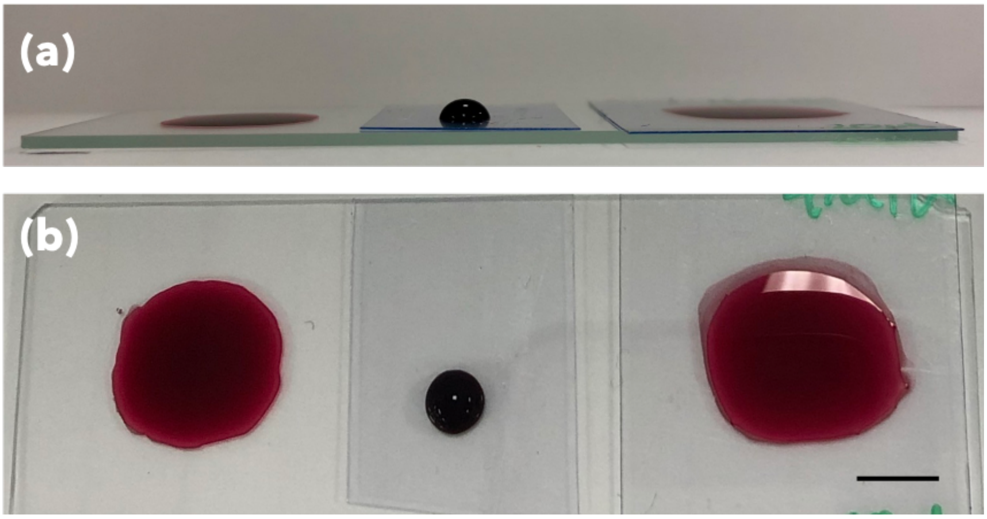

Figure S6. Photograph showing the wettability of **PBI-A** solution on different substrates (left) glass (middle) untreated plastic and (right) plasma treated plastic. Scale bar represents 1 cm.

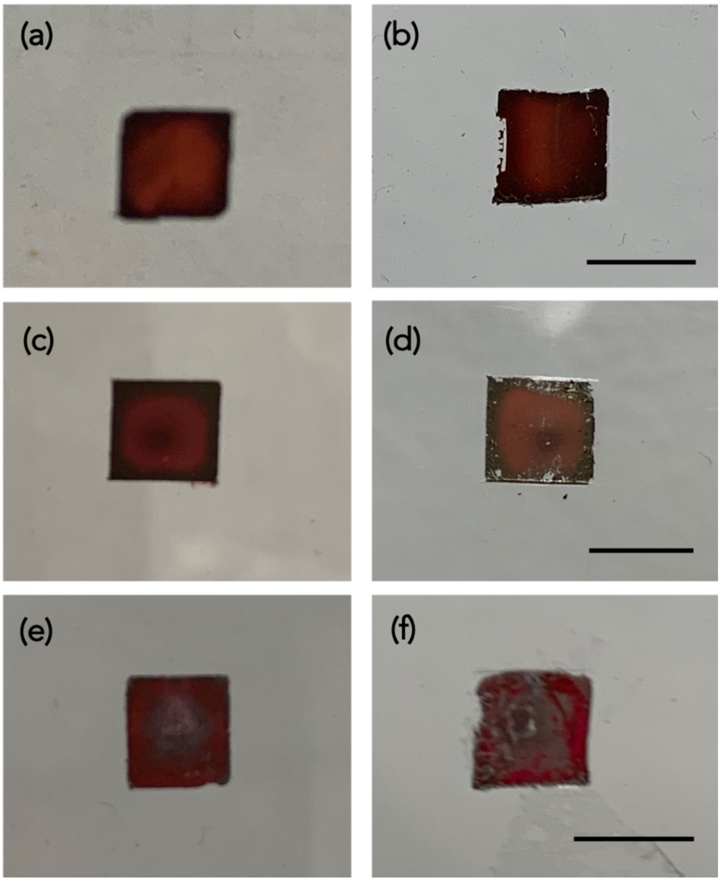

Figure S7. Photographs of films of **PBI-A** ((a) and (b)), **PBI-H** ((c) and (d)) and **PBI-F** ((e) and (f)). (a), (c) and (e) are on glass and (b), (d) and (f) are on treated plastic. The scale bars represent 5 mm.

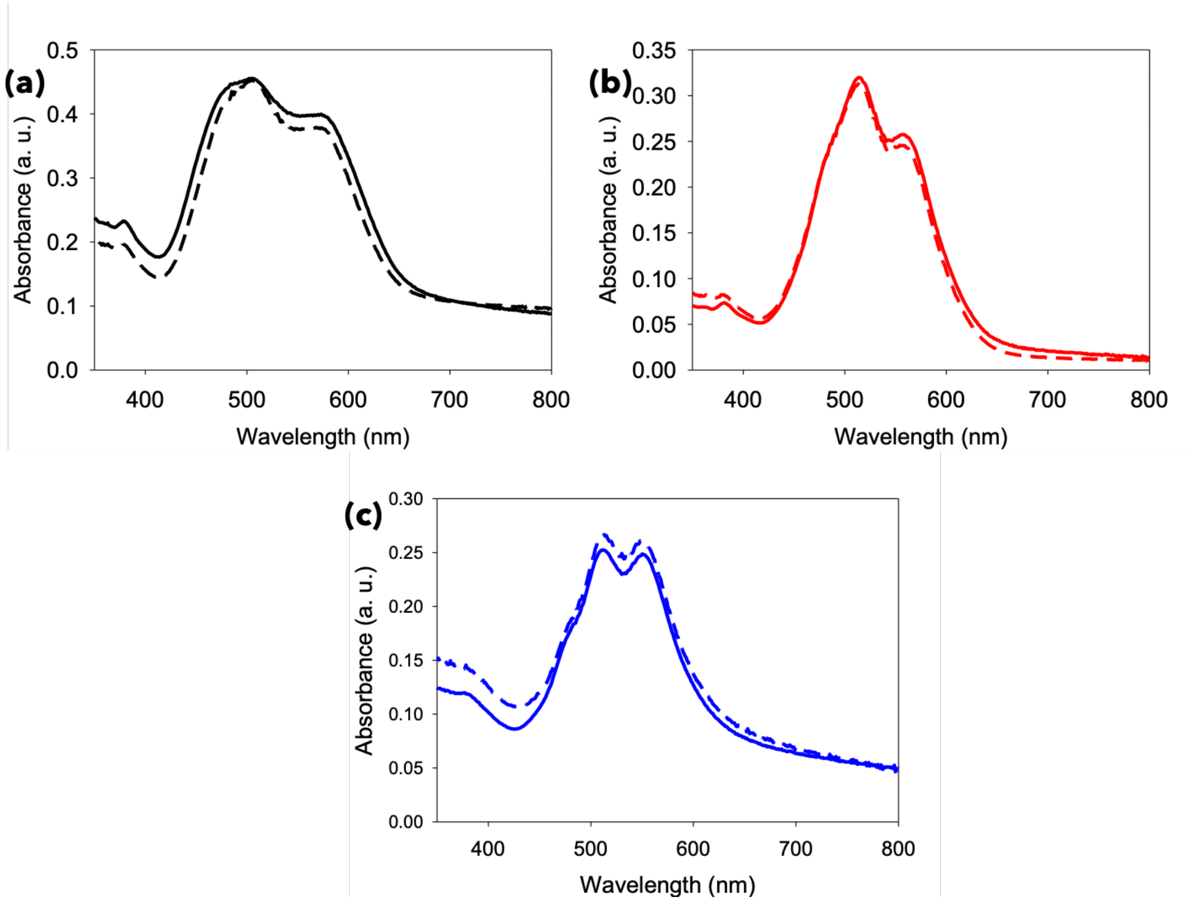

Figure S8. UV-vis absorption data of thin films cast on glass (dashed data) and treated plastic (solid data) for (a) **PBI-A**, (b) **PBI-H** and (c) **PBI-F** showing little difference in the absorption profiles, and therefore aggregation of the molecules in the films.

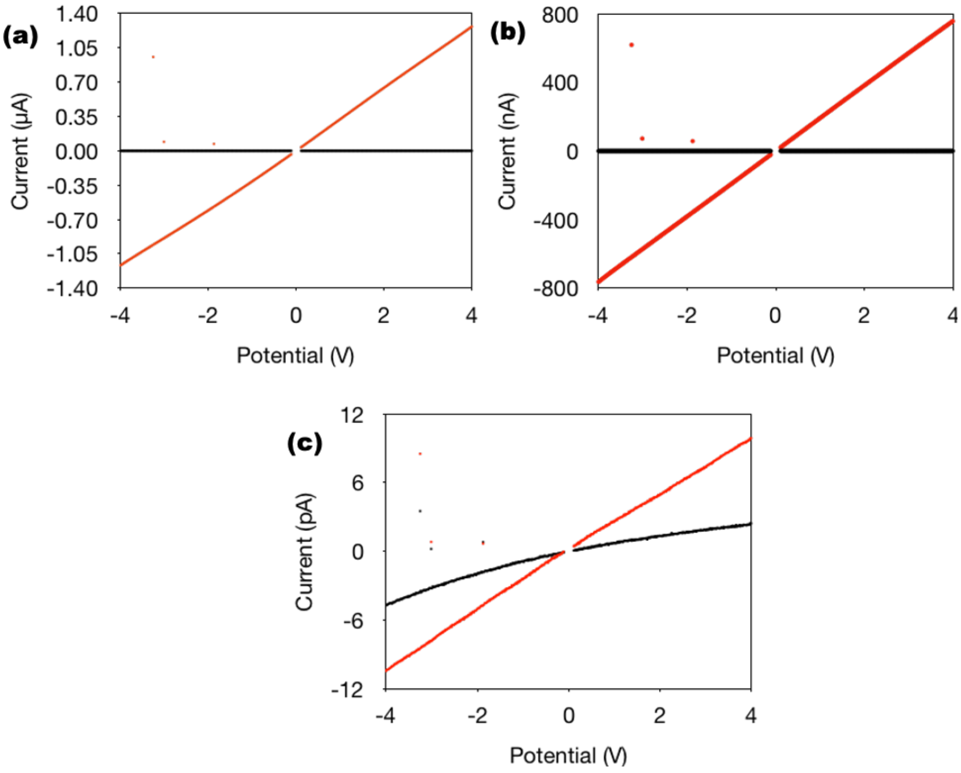

Figure S9. IV traces for films of (a) **PBI-A** (b) **PBI-H** and (c) **PBI-F**. Red is after irradiation with 365 nm LED for 5 minutes.

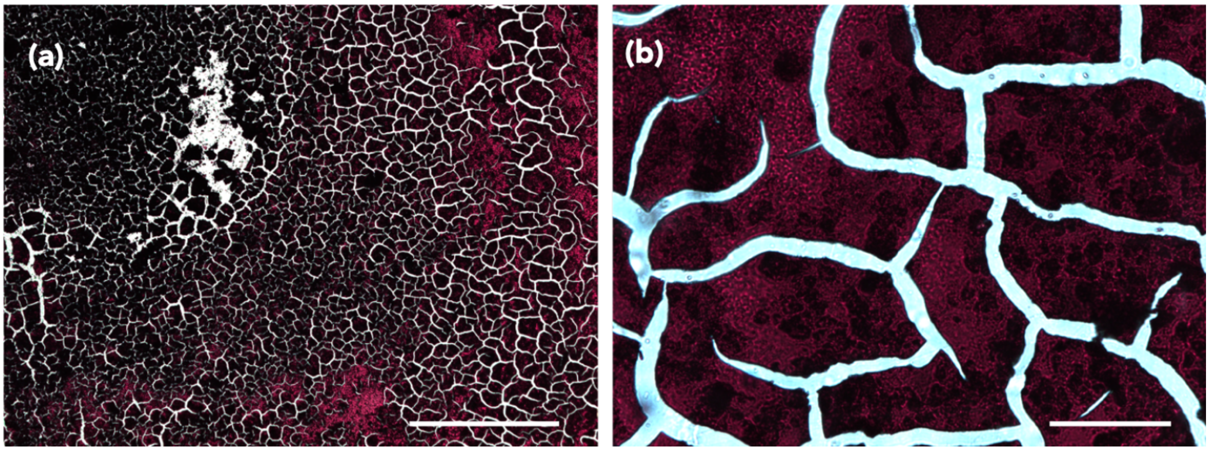

Figure S10. Microscope image of a film of **PBI-F** (a) ×5 and (b) ×50 magnification, showing a cracked surface. The scales bars represent 1 mm and 50 μm respectively.

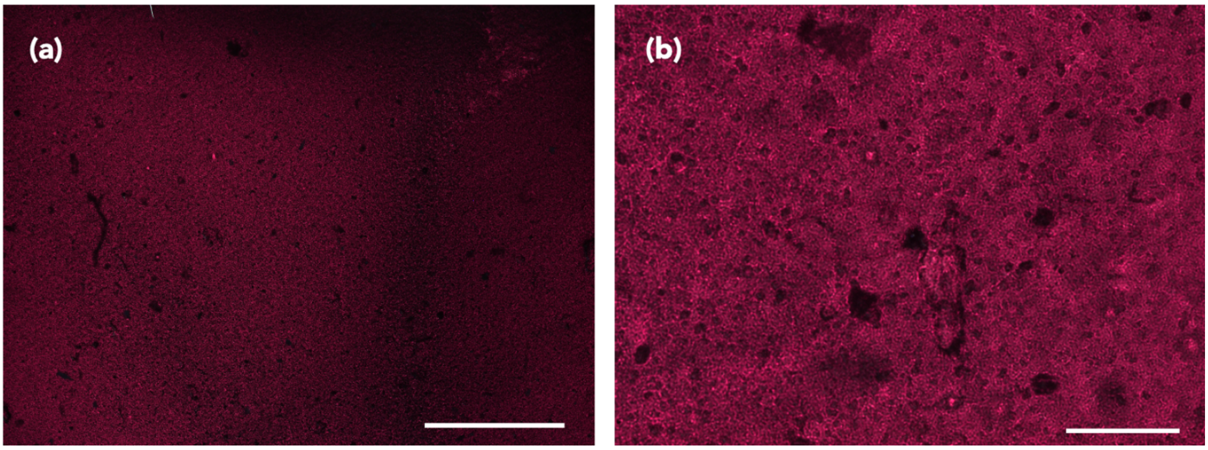

Figure S11. Microscope image of a film of **PBI-A** (a) ×5 and (b) ×50 magnification. The scales bars represent 1 mm and 50 µm respectively.

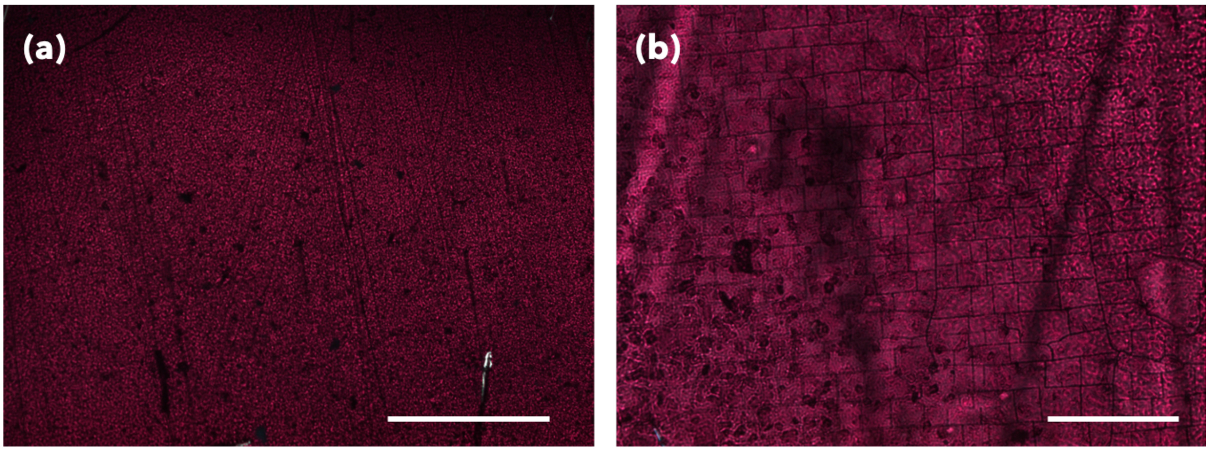

Figure S12. Microscope image of a film of **PBI-H** (a) ×5 and (b) ×50 magnification. The scales bars repronent 1 mm and 50 µm respectivly.

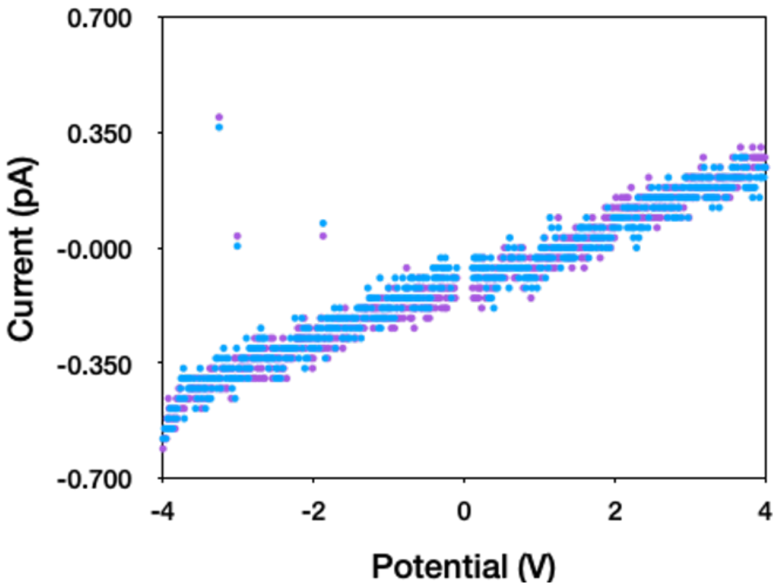

Figure S13. IV curve of a blank sample (no perylene) at 0.0° (blue data) and at 19.1° (purple data) showing no phmic contact and no effect from bending of the substrate alone.

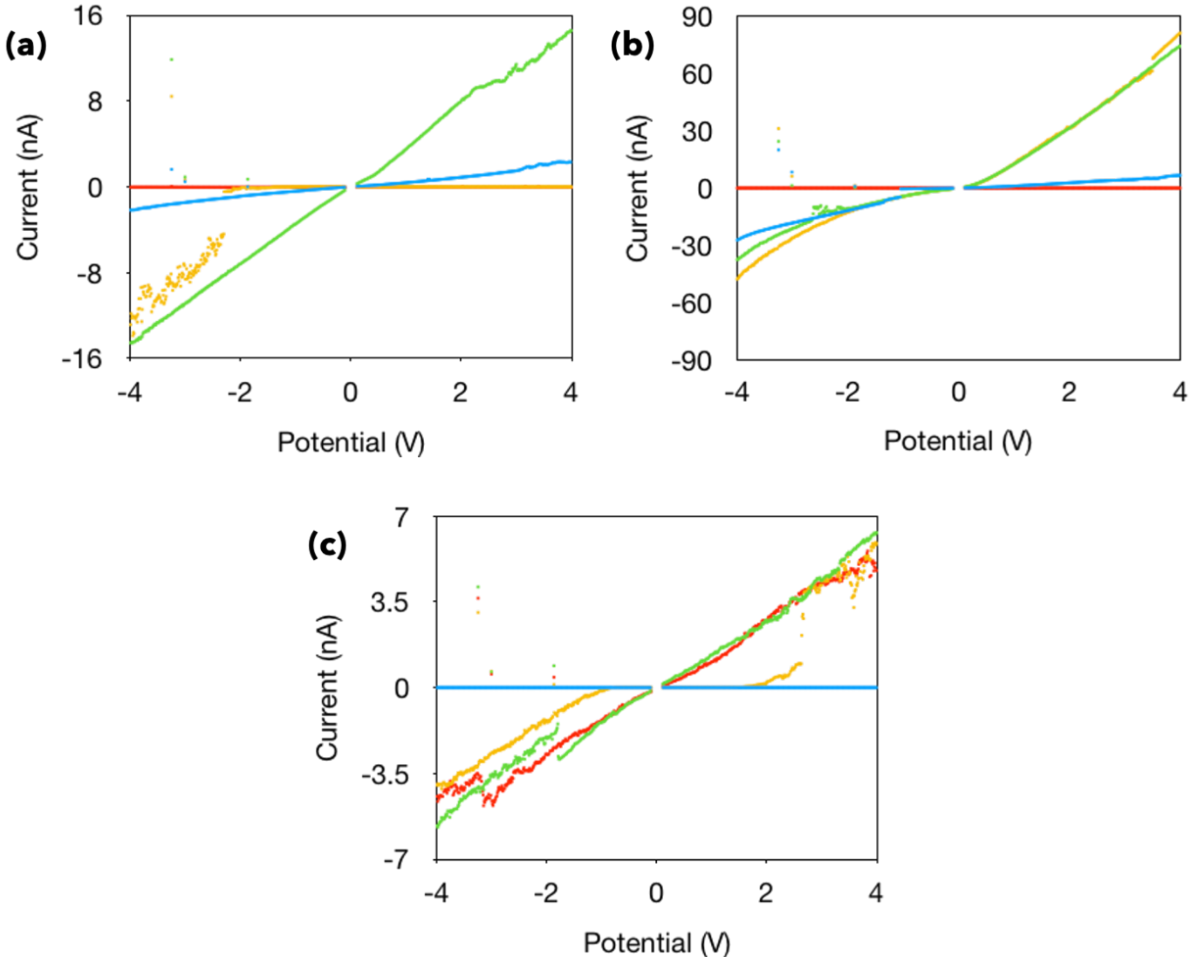

Figure S14. IV curves of repeat films of **PBI-H**. Blue data is flat measurements in the dark and the other coloured data is after the film has been bent under irradiation showing very noisy or no data as the film is damaged from bending. Repeat bending experiments on **PBI-A** films. (a) and (b) show the IV curves for two different films. Blue is at 0.0°, green 9.5°, yellow 11.5°, red 14.3° and purple 19.1°.

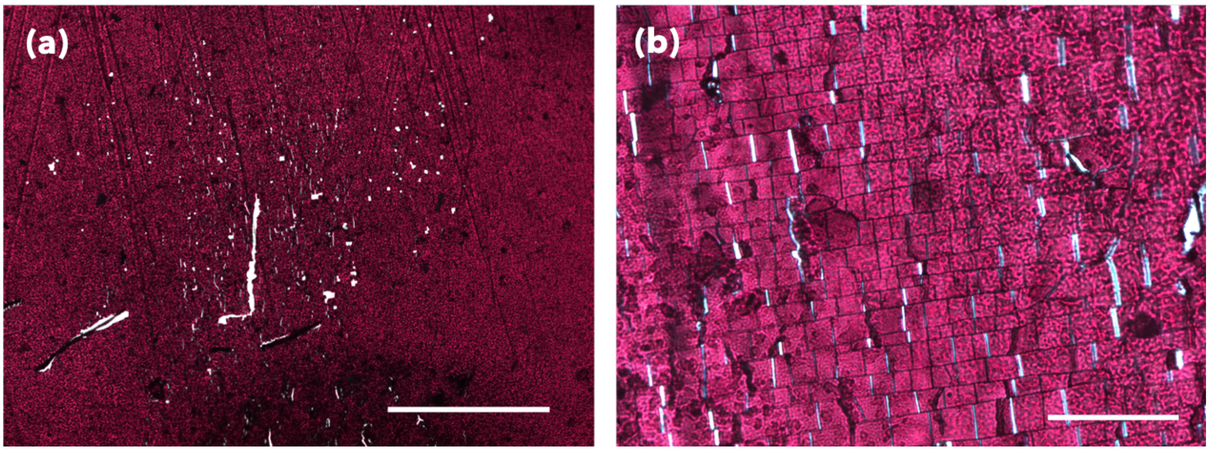

Figure S15. Microscope image of a film of **PBI-H** after being the bending measurements at (a) ×5 and (b) ×50 magnification. The scales bars reprosent 1 mm and 50 µm respectivly.

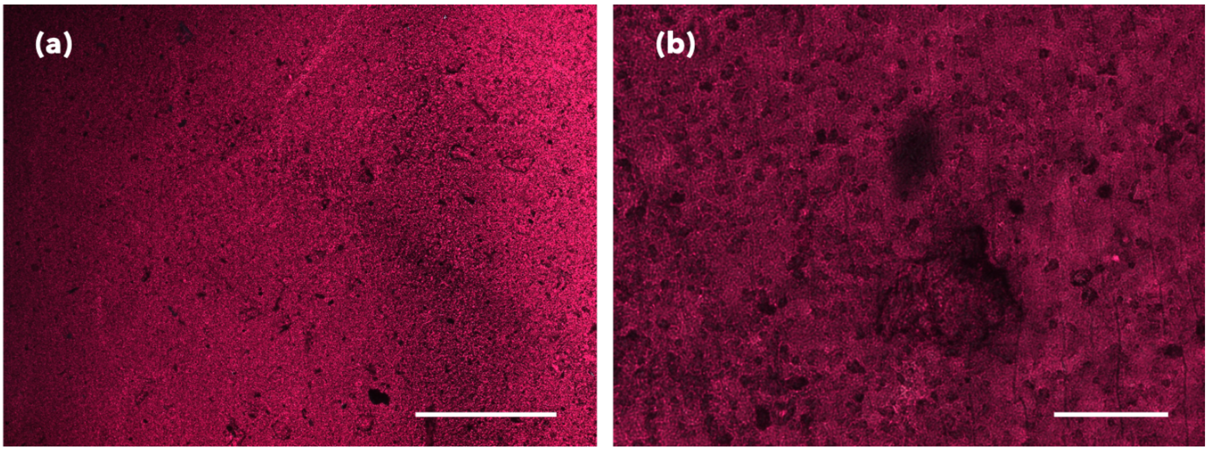

Figure S16. Microscope image of a film of **PBI-A** after the bending experiment (a) ×5 and (b) ×50 magnification. The scales bars represent 1 mm and 50 µm respectively.

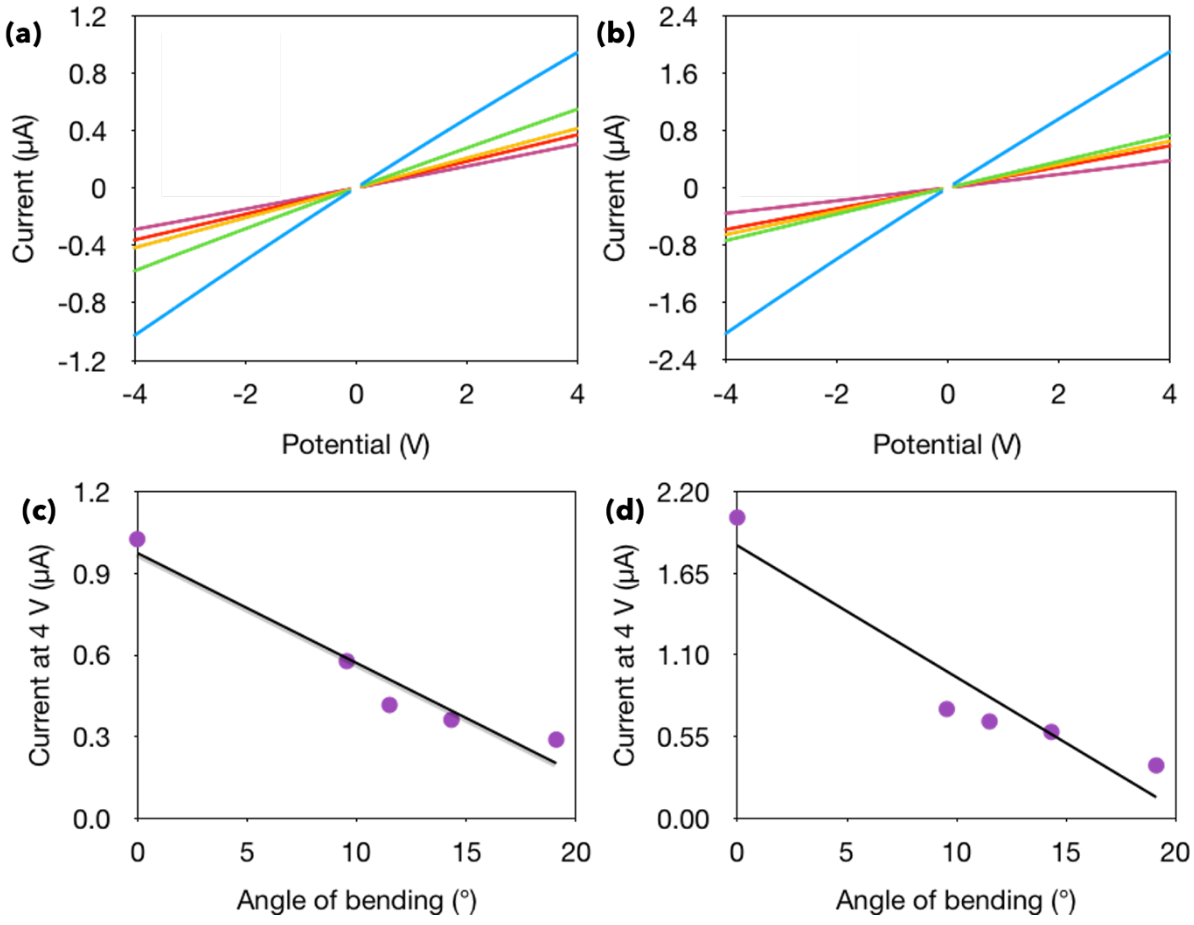

Figure S17. Repeat bending experiments on **PBI-A** films. (a) and (b) show the IV curves for two different films. Blue is at 0.0°, green 9.5°, yellow 11.5°, red 14.3° and purple 19.1°. (c) and (d) show the change in current at 4 V (purple data) and a line of best fit (solid line) for the IV data above it. For (c) R^2 = -0.9701 and (d) R^2= -0.9507.

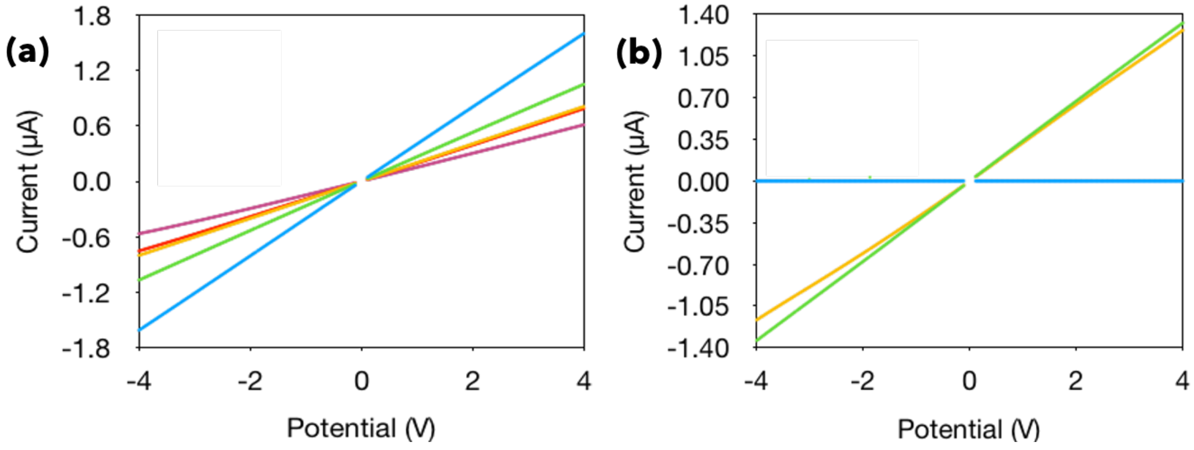

Figure S18. (a) IV data from bending experiments on **PBI-A** films corresponding to Fig. 4 in the main text. (a) and (b) show the IV curves for two different films. Blue is at 0.0°, green 9.5°, yellow 11.5°, red 14.3° and purple 19.1°. (b)IV data for **PBI-A** flat measurement in the dark (blue) and under 365 nm (green) before bending. Then after bending and allowed to relax in the dark (dark blue, can hardly been seen under the other blue data set) and re-irradiated at 365 nm (yellow data).

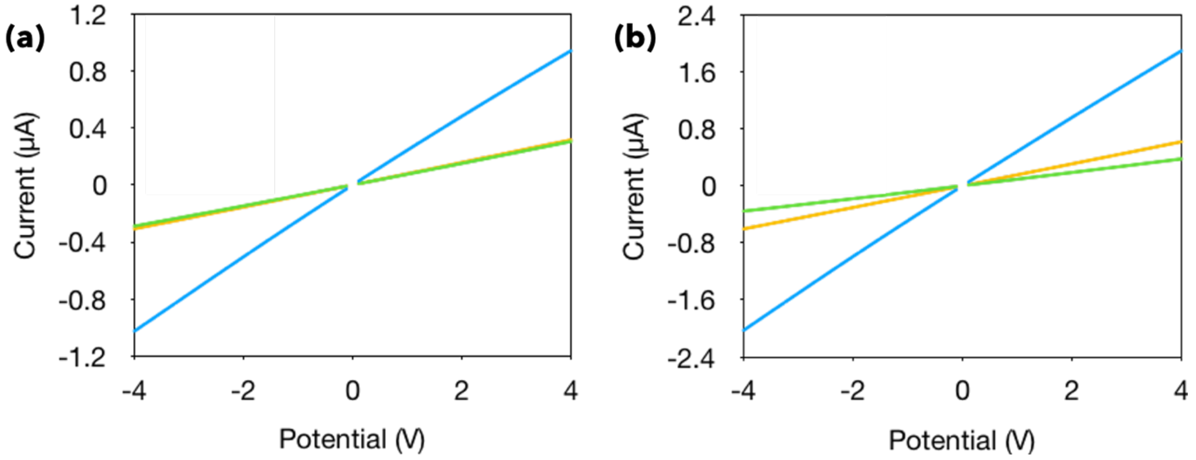

Figure S19. IV data showing the recoverability of the **PBI-A** film, the blue data is at 0°, the green data at 19.1° and the yellow data after the sample was allowed to recover flat in the dark, then remeasured at 19.1°. (a) is for the same film shown in Fig. S17 (a) and (c), and (b) is for the same film shown in Fig. S17 (b) and (d).

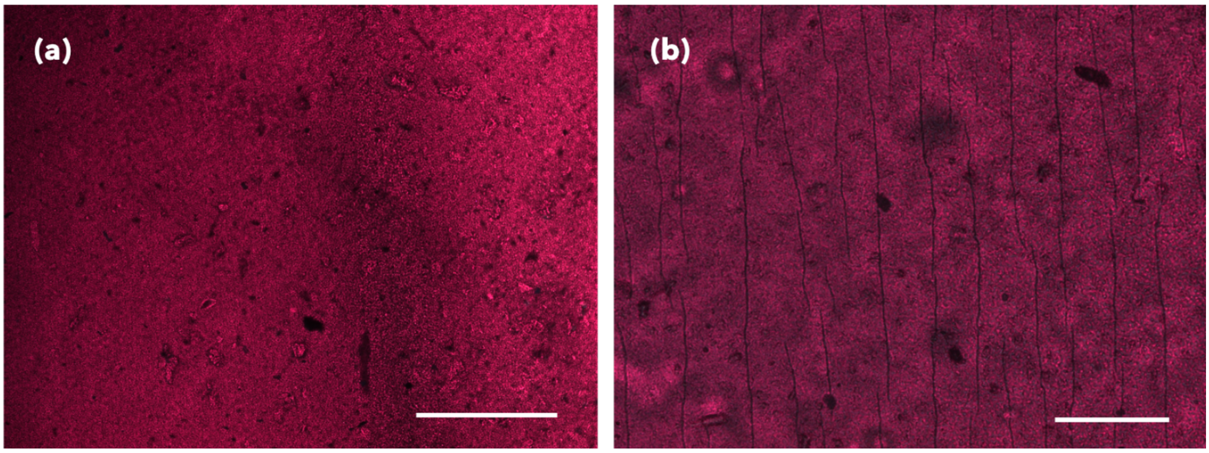

Figure S20. Microscope image of a film of **PBI-A** after the lots of bending by hand (a) ×5 and (b) ×50 magnification. The scales bars represent 1 mm and 50 μm respectively.

References

1. E. R. Draper, J. J. Walsh, T. O. McDonald, M. A. Zwijnenburg, P. J. Cameron, A. J. Cowan and D. J. Adams, 2014, *J. Mater. Chem. C*, **2**, 5570-5575
2. M. Wallace, A. Z. Cardoso, W. J. Frith, J. A. Iggo and D. J. Adams, 2014, *Chem. Eur. J* , **20**, 1 6484 – 1 6487
3. E. R. Draper, M. Wallace, D. Honecker and D. J. Adams, 2018, *Chem. Commun.*, **54**, 10977-10980
4. https://www.openscad.org/downloads.html

5. R. W. Adams, C. M. Holroyd, J. A. Aguilar, M. Nilsson and G. A. Morris, 2013, *Chem. Commun.*, **49**, 358
6. M. Liu, X. Mao, C. Ye, H. Huang, J. K. Nicholson and J. C. Lindon, 1998, *J. Mag. Res.* **132,** 125–129
7. G. R. Fulmer, A. J. M. Miller, N. H. Sherden, H. E. Gottlieb, A. Nudelman, B. M. Stoltz, J. E. Bercaw and K. I. Goldberg, 2010, *Organometallics*, **29**, 2176 2179
8. H. Shinar and G. Navon, 1991, *Biophys J.,* **59**, 203-8.
9. I. Horcas, R. Fernández, J. M. Gómez-Rodríguez, J. Colchero, J. Gómez-Herrero and A. M. Baro, *Rev. Sci. Instrum.*, 2007, **78**, 01735-01735.
